# Utilization of healthcare prior to endometriosis diagnosis: a Danish case–control study

**DOI:** 10.1093/humrep/dead164

**Published:** 2023-08-15

**Authors:** Anna Melgaard, Claus Høstrup Vestergaard, Ulrik Schiøler Kesmodel, Bettina Wulff Risør, Axel Forman, Krina Zondervan, Bodil Hammer Bech, Dorte Rytter

**Affiliations:** Department of Public Health, Research Unit of Epidemiology, Aarhus University, Aarhus C, Denmark; Department of Public Health, Research Unit for General Practice, Aarhus University, Aarhus C, Denmark; Department of Obstetrics and Gynecology, Aalborg University Hospital, Aalborg, Denmark; DEFACTUM, Central Denmark Region, Aarhus N, Denmark; Department of Clinical Medicine, Danish Centre for Health Services Research, Aalborg University, Gistrup, Denmark; Department of Obstetrics and Gynecology, Aarhus University Hospital, Aarhus N, Denmark; Nuffield Department of Women’s and Reproductive Health, John Radcliffe Hospital, Oxford University, Oxford, UK; Department of Public Health, Research Unit of Epidemiology, Aarhus University, Aarhus C, Denmark; Department of Public Health, Research Unit of Epidemiology, Aarhus University, Aarhus C, Denmark

**Keywords:** endometriosis, diagnostic delay, healthcare utilization, women’s health, case–control study

## Abstract

**STUDY QUESTION:**

Do women with endometriosis have higher utilization of primary and secondary healthcare prior to diagnosis compared to women without endometriosis?

**SUMMARY ANSWER:**

Women with a hospital-based diagnosis of endometriosis had an overall higher utilization of both primary and secondary healthcare in all 10 years prior to diagnosis.

**WHAT IS KNOWN ALREADY:**

Endometriosis is associated with a diagnostic delay, but only a few studies have investigated the potential consequences of this delay with regard to the utilization of healthcare. To the best of our knowledge, no study has investigated it in a period corresponding to the estimated diagnostic delay.

**STUDY DESIGN, SIZE, DURATION:**

This national Danish registry-based case–control study included 129 696 women. Cases were women with a first-time hospital-based diagnosis of endometriosis between 1 January 2000 and 31 December 2017.

**PARTICIPANTS/MATERIALS, SETTING, METHODS:**

We identified 21 616 cases using density sampling. Each case was matched on age at the date of diagnosis (index date) to five women without diagnosed endometriosis (n = 108 080). The utilization of healthcare was assessed for the 10 years before the index.

**MAIN RESULTS AND THE ROLE OF CHANCE:**

Cases had significantly higher use of healthcare in all 10 years preceding the index. The mean number of yearly contacts with the GP was 9.99 for cases and 7.85 for controls, with an adjusted incidence rate ratio of 1.28 (1.27; 1.29). For hospital contacts, the association increased slightly in the first 9 years and was most profound in the last year preceding index when the adjusted incidence rate ratio was 2.26 (95% CI 2.28; 2.31).

**LIMITATIONS, REASONS FOR CAUTION:**

We were not able to include women with an endometriosis diagnosis from the general practitioner or private gynaecologist. Therefore, our results are only applicable to hospital-based diagnoses of endometriosis. We do not have information on the specific reasons for contacting the healthcare providers and we can therefore only speculate that the higher utilization of healthcare among cases was related to endometriosis.

**WIDER IMPLICATIONS OF THE FINDINGS:**

This study is in agreement with the other known studies on the subject. Future studies should include specific reasons for contacting the healthcare system and thereby identify any specific contact patterns for women with endometriosis. With this knowledge, healthcare professionals could be better at relating certain healthcare seeking behaviour to endometriosis earlier and thereby reduce the time from onset of symptoms to diagnosis.

**STUDY FUNDING/COMPETING INTEREST(S):**

This study is supported by grants from the project ‘Finding Endometriosis using Machine Learning’ (FEMaLe/101017562), which has received funding from The European Union’s Horizon 2020 research and innovation program and Helsefonden (21-B-0141). K.Z. report grants from Bayer AG, Roche Inc. and Volition, royalties from Oxford-Bayer scientific collaboration in gynaecological therapies, non-financial collaboration with the World Endometriosis Society and World Endometriosis Research Foundation and is a Wellbeing of Women research advisory committee member. All this is outside the submitted work. The other authors have no conflict of interest to declare.

**TRIAL REGISTRATION NUMBER:**

N/A.

## Introduction

Endometriosis is a chronic gynaecological disease, where endometrial tissue is located outside the uterus (World Health Organization, 2018). It is estimated to affect 5–10% of women of childbearing age (Zondervan *et al.*, 2020). The most common symptoms are severe pelvic pain, pain after/during intercourse, painful menstrual periods, fatigue, and infertility, but symptoms can vary and overlap with other gynaecological and gastrointestinal conditions (Zondervan *et al.*, 2020; Becker *et al.*, 2021). The golden standard for diagnosis of endometriosis is through surgical visualization, most commonly laparoscopy (Zondervan *et al.*, 2020). The non-specific symptoms and the invasive diagnostic procedure contribute to a severe underdiagnosis of the condition (Agarwal *et al.*, 2019; Zondervan *et al.*, 2020).

In addition to being underdiagnosed, endometriosis is associated with a diagnostic delay, that has been reported to be as long as 10 years (Hadfield *et al.*, 1996; Husby *et al.*, 2003; Hudelist *et al.*, 2012; Staal *et al.*, 2016; Ghai *et al.*, 2020). The reason for this delay is most likely multifactorial. Women can have difficulties distinguishing between normal and abnormal symptoms and therefore do not seek timely medical help (Shadbolt *et al.*, 2013; Randhawa *et al.*, 2021). Also, physicians may have insufficient knowledge about endometriosis and may tend to normalize symptoms, which can cause incomplete examinations, a lack of referral or referrals to the wrong specialist (Moradi *et al.*, 2014; van der Zanden *et al.*, 2018; Ghai *et al.*, 2020; Roullier *et al.*, 2021).

Few studies have previously investigated the potential consequences of this diagnostic delay on healthcare use. In a study by Surrey *et al.* (2020) using data from the US Optum Research Database, they found that the utilization of all-cause and endometriosis-related healthcare 5 years prior to diagnosis increased with a longer diagnostic delay. Another study by Ballard *et al.* (2008) using data from the UK General Practice Research Database found, that in the 3 years before diagnosis, women with endometriosis consulted their general practitioner (GP) more frequently and with significantly more symptoms related to endometriosis (e.g. dysmenorrhoea, infertility/subfertility, ovarian cysts, etc.) compared to women without endometriosis.

To increase our knowledge of the potential consequences of the diagnostic delay, this national registry-based study aimed to describe the utilization of primary and secondary healthcare in the 10 years preceding diagnosis among women with a hospital-based diagnosis of endometriosis and to compare it to a group of age-matched women without diagnosed endometriosis.

## Materials and methods

### Study population and design

The study was conducted as a national Danish registry-based case–control study with density sampling. All Danish citizens are assigned a unique civil personal registration number (CPR number), which was used to identify the source population. Women entered the source population on 1 January 2000, on their 15th birthday or when they had lived in Denmark for at least consecutive 10 years, whichever came last. Women left the source population on 31 December 2017, their 55th birthday, their date of immigration, death, or date of receiving a first-time hospital-based diagnosis of endometriosis, whichever came first. Cases were identified as women who received a first-time hospital-based diagnosis of endometriosis during the study period. Women with a first-time diagnosis before the study period were identified and excluded. The date of diagnosis for cases was the index, and each case was matched on birth year with five controls who did not have a diagnosis of endometriosis at the time of matching.

### Endometriosis diagnosis

Information on endometriosis diagnosis was obtained from the Danish National Patient Registry which contains information on all public hospital contacts and diagnoses from 1977 and for all private hospital contacts and diagnoses from 2002 (Schmidt *et al.*, 2015). We identified cases with a first-time recorded hospital-based diagnosis of endometriosis (ICD-8 codes 62530 and 62532–62539 from 1977 to 1994 and ICD-10 codes DN801–809 from 1994 onwards). The date of first hospital contact with a diagnosis of endometriosis was included as the date of diagnosis and we included both A and B diagnoses; an A-diagnosis was where endometriosis was the main reason for hospital contact and B-diagnosis was where it was not the main reason for contact. We only had access to information on endometriosis diagnosed in a hospital setting. Endometriosis diagnosed by a private gynaecologist is not included.

For a sub-analysis, we included information on women with a histologically verified endometriosis diagnosis from The National Pathology Data bank, which contains information on pathoanatomical tests (The National Pathology Data Bank, 2022). We identified women with endometriosis (SNOMED-code M76500, M33530, and M76510), corresponding to ∼60% of all cases.

### Healthcare utilization

In Denmark, all Danish citizens have free access to the healthcare system with direct access to a GP. The GP works as a gatekeeper for all specialists, e.g. private gynaecologists and hospital care (except emergency contacts) (Christiansen, 2002; Pedersen *et al.*, 2012).

We included primary and secondary healthcare utilization 10 years preceding the index time. However, only healthcare utilization from 10 years of age and up was included, meaning that women who were between 15 and 19 years of age at index (4% of the study population) only contributed with 5–9 years of information. This was to ensure that the utilization of healthcare could somehow be related to endometriosis, which was only relevant after the women entered puberty (Brix *et al.*, 2019). We only included whole calendar years.

#### Primary healthcare utilization

Information on the number and type of contact with the GP and private gynaecologists was obtained from the Danish National Health Service Register, which contains information on all services provided in the primary healthcare system from 1990 and onwards (Andersen *et al.*, 2011). For GP contacts, we differentiated on type of contact (face-to-face, telephone, email, and home-visits) and whether it was daytime (weekdays between 8 and 16) or out-of-hours. Furthermore, we included para-clinical tests provided by the daytime GP, such as blood samples, urine tests, and C-reactive protein (CRP) test. We included rapid streptococcal test (strep-A) as a negative control, as this was not considered to be associated with endometriosis. For the private gynaecologist, we included face-to-face and telephone contacts.

#### Secondary healthcare utilization

Information on the number and any type of hospital contact (inpatient, outpatient and emergency (only for public hospitals)) was obtained from the Danish National Patient Registry. Information on contacts with psychiatric departments was only in the registry from 1995 and onwards. For private hospitals, the registry only holds information from 2002, but the majority of patients are managed in the public healthcare system (98.75% of all contacts in 2017) (Holstein, 2019).

### Covariates

We used the CPR number to link data from the included Danish Registers. Information on date of birth, region of residence, ethnicity (Danish, immigrant, or descendant of an immigrant) household composition (single, couples, or other), and migration in and out of Denmark was obtained from the Danish Civil Registration System (Erlangsen and Fedyszyn, 2015).

Information on levels of highest completed education (primary, upper secondary, short cycle tertiary or BA, masters or equivalent, or PhD) and socioeconomic status (self-employed/executive, employed, on social benefits, student, or other) was obtained from Statistics Denmark’s register for education and family income. For the socioeconomic status, we used the highest status for the household which also included children under 25 years of age living at home. Information on socioeconomic status was first recorded from 1994 and forward, therefore we used information from 1994 as a substitute for the years 1990–1993 under the assumption that socioeconomic status is unlikely to change rapidly or substantially. For women aged 15–25, we used information about the educational level from the parent with the highest educational level under the assumption that the parent’s educational level had a higher impact on healthcare utilization behaviour when the woman was young.

Information on parity (0, 1, or ≥2 children) was obtained from The Medical Birth Registry.

### Statistical analyses

The distribution of characteristics for cases and controls was presented with numbers and percentages.

To investigate the association between utilization of primary and secondary healthcare among cases and controls, we calculated the mean number of yearly contacts with the GP, private gynaecologist and hospital for the entire study period. This was also estimated for each type of contact (e.g. face-to-face, telephone), paraclinical tests provided by the GP, as well as inpatient, outpatient and emergency hospital contacts. Using negative binomial regression analysis, we estimated crude and adjusted incidence rate ratios (IRRs) and 95% CI for the entire study period. IRRs were adjusted for the region of residence, educational level, house type, socioeconomic status, ethnicity, parity and age (matching variable). For each covariate, missing values were included in a separate group. Information for adjustments was extracted 1 year before the index date. Cluster robust variance was applied to account for the cluster correlation between contacts within the same woman.

To investigate the development in healthcare utilization over the 10 years preceding the index date, we calculated the yearly mean number of GP daytime-, out-of-hour-, and hospital contacts, respectively, for cases and controls. Furthermore, we estimated the yearly adjusted IRRs and 95% CI. Information on time-varying covariates (all but ethnicity) was extracted yearly from the registers and adjusted for.

An additional *post hoc* analysis was performed in order to estimate the adjusted IRRs and 95% CIs for GP daytime contacts, out-of-hour contacts, and hospital contacts in the 1 year following the index to investigate whether the diagnosis led to a reduction in healthcare utilization in that year.

Since the negative control analysis indicated an association between endometriosis and number of strep-A tests performed, a *post hoc* analysis was conducted to investigate whether the proportion of positive strep-A tests among tested women was the same among cases and controls. We defined a strep-A test to be positive if a prescription of antibiotics (ATC code J01) was redeemed within 7 days after the test was performed. Information on redemptions was retrieved from the Danish National Prescription Registry.

In a sub-analysis, we restricted the analysis to only include cases with a histologically verified diagnosis of endometriosis and their respective controls.

All statistical analyses were performed in Stata 17 (StataCorp. 2021. College Station, TX, USA: StataCorp LLC).

### Ethics

The study was approved by the Danish Data Protection Agency under the Aarhus University comment agreement and Aarhus University j.number 2016-051-000001, sequential number 1242 (Date: 27 September 2018). According to Danish legislation, ethical approval of registry studies is not required.

## Results

We identified 21 616 women who had a first-time hospital-based diagnosis of endometriosis at some point between 1 January 2000 and 31 December 2017. Among these cases, 12 728 (58%) had a histologically verified diagnosis. The mean age at diagnosis was 34.6 (8.9) years. The prevalence of hospital-based diagnosed endometriosis among women aged 15–55 was 1.64% in 2017.

The distribution of included characteristics among cases and controls are presented in Table 1. There were no marked differences in educational level, household type, or socioeconomic status between cases and controls. However, there were higher numbers of cases living in the North Denmark Region, Central Denmark Region, Region of Southern Denmark compared with controls, and cases were more likely to have Danish origin. Also, as expected given the association between endometriosis and infertility, a larger proportion of cases did not have children.

**Table 1.** Characteristics of women with hospital-based diagnosed endometriosis (cases) and age-matched women without endometriosis (controls) with numbers and percentages.

Characteristics	**Cases (n = 21** **616)**	**Controls (n = 108** **080)**
Age at matching, mean (SD)	34.6 (8.9)	34.6 (8.9)
Age at matching, n (%)		
15–24	3770 (17.4)	18 861 (17.5)
25–34	7935 (36.7)	39 624 (36.7)
35–44	7086 (32.8)	35 529 (32.9)
45–55	2825 (13.1)	14 066 (13.0)
Year of diagnosis, n (%)		
2000–2002	3430 (15.9)	
2003–2005	3916 (18.1)	
2006–2008	3652 (16.9)	
2009–2011	3725 (17.2)	
2012–2014	3493 (16.2)	
2015–2017	3400 (15.7)	
Region of residence, n (%)		
North Denmark region	3073 (14.2)	10 981 (10.2)
Central Denmark region	5390 (24.9)	24 315 (22.6)
Region of Southern Denmark	4603 (21.3)	22 277 (20.7)
Capital region	5845 (27.0)	34 898 (32.4)
Region Zealand	2699 (12.5)	15 124 (14.1)
Missing	6 (0)	485 (0.4)
Highest educational level, n (%)		
Primary	3902 (18.6)	20 092 (19.3)
Upper secondary	9692 (46.1)	47 172 (45.4)
Short cycle tertiary or BA	5835 (27.8)	27 650 (26.6)
Master or equivalent	1526 (7.3)	8562 (8.2)
PhD	69 (0.3)	488 (0.5)
Missing	592 (2.7)	4116 (3.8)
Household type, n (%)		
Single	4780 (22.1)	24 276 (22.6)
Couples	14 475 (67.0)	70 776 (65.8)
Other	2355 (10.9)	12 543 (11.7)
Missing	6 (0)	485 (0)
Socioeconomic status, n (%)		
Self-employed/executive	1593 (7.4)	8604 (8.0)
Employed	16 843 (77.9)	80 222 (74.6)
Social benefit	1834 (8.5)	10 467 (9.7)
Student	1137 (5.3)	7148 (6.6)
Other*	(≤1)	1153 (1.1)
Missing	(≤1)	486 (0.4)
Ethnicity, n (%)		
Danish	20 350 (94.2)	100 161 (92.7)
Immigrant	1054 (4.9)	6372 (5.9)
Descendant of immigrant*	(≤1)	1461 (1.4)
Missing	(≤1)	86 (0.1)
Parity, n (%)		
No children	15 710 (72.7)	67 998 (62.9)
1 child	2055 (9.5)	10 856 (10.0)
2 or more children	3851 (17.8)	29 226 (27.0)

Characteristics were extracted from registers 1 year before index with exception of age, which was defined at index.

* Due to few observations in some categories, some numbers and percentages have been concealed.

### Primary healthcare utilization

The majority of the included contacts in primary healthcare were with the GP during daytime, where cases had an average of 9.99 contacts, and controls had 7.85 contacts per year. Table 2 shows, that cases on average had 26% more yearly contacts with the GP during daytime hours (IRR 1.26% (95% CI 1.25; 1.28)) and 43% more out-of-hour GP contacts (IRR 1.43% (95% CI 1.39; 1.46)). The significantly higher number of contacts among cases was applicable for all types of contacts and all paraclinical tests included in the analyses. The average number of yearly contacts with the private gynaecologists was generally low compared to number of GP-contacts, but cases had more contacts than controls (IRR 2.22 (95% CI 2.05; 2.39)).

**Table 2.** Association between healthcare utilization and hospital-based diagnosed endometriosis presented by the mean number of contacts per year, crude incidence rate ratios (IRR), adjusted IRR, and 95% CI in the 10 years preceding index.

	Cases	Controls	Crude IRR	Adjusted IRR
**Primary healthcare utilization**				
All GP-contacts	9.99	7.85	1.27 (1.26; 1.29)	1.28 (1.27; 1.29)
Daytime GP-contacts total	8.37	6.66	1.26 (1.24; 1.27)	1.26 (1.25; 1.28)
Face-to-face	4.67	3.79	1.23 (1.22; 1.25)	1.24 (1.23; 1.25)
Telephone	3.35	2.61	1.28 (1.27; 1.30)	1.28 (1.27; 1.30)
Email	0.34	0.25	1.36 (1.30; 1.42)	1.45 (1.38; 1.53)
Home visits	0.01	0.01	1.19 (1.03; 1.37)	1.26 (1.11; 1.43)
Urine sample	0.37	0.27	1.40 (1.37; 1.43)	1.39 (1.36; 1.42)
Blood sample	0.42	0.32	1.31 (1.29; 1.34)	1.29 (1.27; 1.31)
C-reactive protein (CRP) test	0.18	0.12	1.51 (1.47; 1.55)	1.55 (1.51; 1.59)
Rapid streptococcal test (strep-A)	0.16	0.14	1.18 (1.16; 1.21)	1.20 (1.18; 1.22)
Out-of-hour contact total	0.48	0.35	1.36 (1.33; 1.40)	1.43 (1.39; 1.46)
Face to face	0.28	0.19	1.47 (1.43; 1.51)	1.44 (1.41; 1.48)
Telephone	0.17	0.14	1.22 (1.18; 1.27)	1.38 (1.34; 1.43)
Home visits	0.03	0.02	1.34 (1.21; 1.48)	1.52 (1.40; 1.65)
All private gynaecologists contact	0.03	0.01	2.30 (2.14; 2.47)	2.22 (2.05; 2.39)
**Secondary healthcare utilization**				
All hospital contacts	1.21	0.89	1.37 (1.34; 1.39)	1.39 (1.37; 1.41)
Inpatient	0.28	0.22	1.26 (1.23; 1.28)	1.28 (1.26; 1.31)
Outpatient	0.71	0.50	1.43 (1.41; 1.45)	1.45 (1.43; 1.47)
Emergency	0.22	0.16	1.33 (1.29; 1.36)	1.37 (1.34; 1.40)

Data were adjusted for the region of residence, educational level, household type, socioeconomic status, parity, and ethnicity. Information on covariates was extracted from registers at 1 year before the index.

**Figure 1.**
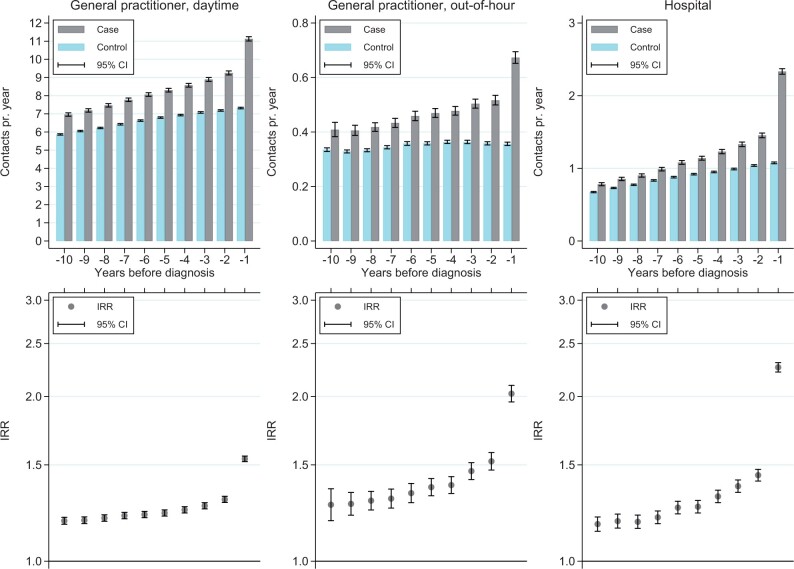
Yearly mean number of any daytime and out-of-hour contacts with the general practitioner and hospital contacts in the 10 years preceding index for cases and controls, and yearly incidence rate ratios (IRRs) with 95% CI.

Figure 1 shows, that 10 years prior to the diagnosis of endometriosis, cases had 19% (IRR 1.19 (95% CI 1.17; 1.20)) more daytime GP contacts compared to controls. This association was stable but with an increase starting 4 years before the index and was most profound in the last year, with the IRR reaching 1.54 (95% CI 1.52; 1.56). There was a similar association for out-of-hour GP contacts, however with a more profound increase in the last year before the index (IRR 2.02 (95% CI 1.96; 2.10)).

The *post hoc* analysis showed, that in the 1 year following the diagnosis, the IRR for daytime GP contacts showed a minor decrease to 1.46 (95% CI 1.45; 1.49) and for out-of-hour GP the IRR decreased slightly to 1.81 (95% CI 1.75; 1.88).

For the negative control analyses, cases on average had 20% more strep-A tests performed each year (IRR 1.20 (95% CI 1.18; 1.22)). This was applicable for all 10 years prior to index. Among controls, 27.1% of tests performed were positive. Among cases, this proportion was 25.9%. Hence, cases had more tests performed, but had a 4% lower risk of the test being positive.

### Secondary healthcare utilization

The average number of any hospital contact per year was 1.21 for cases and 0.89 for controls. Cases had 28% more inpatient contacts (IRR 1.28 (95% CI 1.26; 1.31)), 45% more outpatient (IRR 1.45 (95% CI 1.43; 1.47)), and 37% more emergency contacts (IRR 1.37 95% CI (1.34; 1.40)) per year, compared to controls (Table 2). Figure 1 shows, that in the 10-year period, controls on average had 1 or fewer hospital contacts per year. For cases, the mean yearly number of contacts increased slightly over the first 9 years, with a more profound increase in the last year preceding index, leading to an IRR of 2.26 (95% CI 2.28; 2.31).

The post-hoc analysis showed an increase in the IRR for hospital contacts in the year following diagnosis to 2.69 (95% CI 2.64; 2.74).

### Histologically verified diagnosis of endometriosis

**Figure 2.**
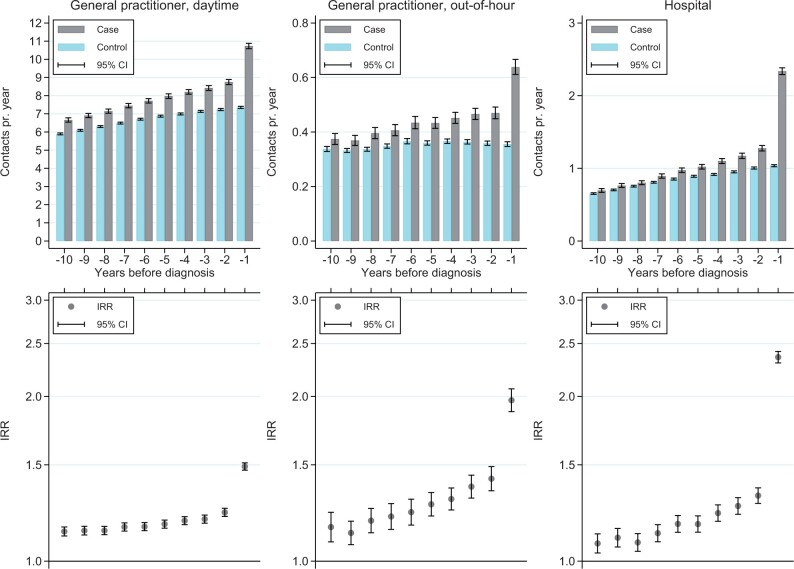
Yearly mean number of any daytime and out-of-hour contacts with the general practitioner and hospital contacts in the 10 years preceding index for cases with a histologically verified diagnosis of endometriosis and their respective controls, and yearly incidence rate ratios (IRRs) with 95% CI.

In the sub-analyses only including women with a histologically verified diagnosis and their controls, the same overall associations were found, with significantly more GP daytime and out-of-hour contacts as well as hospital contacts among cases compared with controls, although the IRRs were generally slightly lower compared with the main analyses (Fig. 2).

## Discussion

This nationwide case–control study showed that women with hospital-based diagnosed endometriosis had significantly higher utilization of primary and secondary healthcare 10 years prior to diagnosis compared to age-matched women without diagnosed endometriosis. This was applicable for each of the 10 years, but the largest difference was in the last years leading up to the diagnosis.

In this study, we found a prevalence of hospital-based diagnosed endometriosis to be 1.64% in 2017. A previous study found that the incidence of hospital-based diagnosed endometriosis in Denmark increased from 1990 to 2017, starting with an incidence rate of 5.46 (95% CI 5.07; 5.87) per 10 000 person-years and rising to an incidence rate of 8.01 (95% CI 7.50; 8.56) per 10 000 person-years (Illum *et al.*, 2022).

The results showed that women with endometriosis on average had 28% more yearly contacts with the GP compared to women without endometriosis. This association corresponds well to findings from a study from the UK, where Ballard *et al.* investigated patient-reported symptoms and consultation patterns at the GP 3 years before receiving an endometriosis diagnosis. They found that women with endometriosis consulted the GP more than twice as often in the 3 years before diagnosis compared to women without endometriosis (Ballard *et al.*, 2008). The differences in our estimates might be caused by differences in case status, as we only included hospital-based diagnosed cases, whereas Ballard *et al.* also included cases with a diagnosis code for endometriosis registered by the GP. It could also be caused by differences in healthcare systems or different healthcare seeking behaviours. The study by Ballard also found an increase in the number of contacts in the year leading up to diagnosis, supporting our findings. In our study, we were not able to include the reason for consulting the GP, as the Danish registers do not hold this information. However, Ballard *et al.* (2008) found that symptoms related to endometriosis was a relatively uncommon reason for contact among women without endometriosis.

In the present study, we found that women with endometriosis on average had 43% more yearly contacts with the out-of-hour GPs compared to controls. The reasons for out-of-hour contacts are often more acute and severe than during the daytime. This could indicate that women with endometriosis, in addition to chronic pain, also more often suffer from acutely emerging and severe symptoms.

We found that cases had more urine samples taken than controls. This is supported by the study of Ballard *et al.* where they found that women with endometriosis had more urinary symptoms such as cystitis (OR 1.7 (1.5; 1.9)), urinary tract infection (OR 2.1 (2.0; 2.3)), and dysuria (OR 2.4 (2.1; 2.7)) in the 3 years before diagnosis. Furthermore, we found that cases had more blood samples and CRP tests provided by the GP. This could either indicate that cases have more infections or that the GP performed these tests because the reported symptoms suggest infection or symptoms that needed more clarification.

We included strep-A tests as a negative control in our analysis, as we believed that having a strep-A test performed, would be unrelated to endometriosis. Surprisingly, we found that cases had a significantly higher number of strep-A tests performed, and this was evident already 10 years before receiving an endometriosis diagnosis. A potential explanation for this association, could be more frequent healthcare seeking behaviour in women with endometriosis. Hence, cases might have a lower threshold for contacting the GP and may therefore also be more likely to have a strep-A test performed. However, looking at indicators for a positive test as described in ‘Materials and methods’ section, we found that cases and controls had approximately the same probability of a positive test result among those tested. This could indicate that cases and controls had the similar thresholds for contacting the GP when experiencing symptoms related to a strep-A infection. The difference in frequency of strep-A tests performed among cases and controls is therefore less likely to be explained by differences in healthcare seeking behaviour. Another explanation could be that women with endometriosis are more likely to have infections, however, to our knowledge, there is no research indicating this association.

The higher utilization of hospital care already several years before the index for cases compared to controls found in the present study, are supported by a study by Surrey *et al.* They found that the mean number of ambulatory visits, emergency room visits, and inpatient stays in the pre-diagnosis period increased with the length of the diagnostic delay when comparing a short delay (≤1 year), intermediate delay (1–3 years) and long delay (3–5 years) (Surrey *et al.*, 2020). As information on the exact time for onset of endometriosis symptoms is unavailable to us, we were unable to calculate the individual diagnostic delays. The higher number of contacts among cases compared to controls already 10 years before diagnosis could indicate that symptoms of endometriosis was present long before receiving a diagnosis and hence be an indicator of diagnostic delay. However, the higher number of contacts could also indicate that women with endometriosis have other conditions or comorbidity that causes them to seek healthcare.

In the *post hoc* analysis, we found a slight decrease in the IRRs for contacts with the GP during daytime and out-of-hour in the 1 year following diagnosis compared to the year leading up to diagnosis. However, we found an increase in hospital contacts. The increase could be explained by follow-up consultations at the hospital after diagnosis and/or follow-up consultations or complications after surgery.

The study of Ballard *et al.* found a stronger association among women with a definite diagnosis of endometriosis and endometriosis symptoms reported to the GP 3 years before diagnosis, when they stratified cases by diagnosis certainty (‘definite’, ‘probable’, and ‘possible’ cases) (Ballard *et al.*, 2008). In our sub-analysis, we only included cases with a histologically verified diagnosis. The results showed a slightly attenuated association compared to the main analysis. The additional contacts in histologically verified cases are, however, more likely explained by endometriosis symptoms. Of all included cases, 58% had a histologically verified diagnosis, indicating that the remaining cases were diagnosed based on symptoms and/or visualization. From a clinical perspective, the percentages of cases with a histologically verified diagnosis do not seem unreasonable in a Danish context. However, the diagnostic process and method may vary between countries and healthcare systems (Becker *et al.*, 2021).

### Strengths and limitations

A major strength of our study is that we used information from the Danish national registers, where it is possible to include the entire population and link all data from the included registers at the individual level. Thereby we have a well-defined population at risk. Information on healthcare utilization is based on administrative data, hence there is no reason to believe that the quality of data should depend on diagnosis status. It is also a strength of our study that we performed our main analysis on the entire study population and restricted the sub-analysis to women with a histologically verified and thereby more certain diagnosis of endometriosis.

Furthermore, we included both primary and secondary healthcare and thus all contacts related to non-severe and severe symptoms. Since the GPs work as gatekeepers and are needed for referral for hospital care, the contacts with the hospital might be less sensitive to healthcare seeking behaviour. Therefore, our results for primary and secondary healthcare support and strengthen each other.

A limitation is that even though we included all types of hospital-diagnosed endometriosis, we were not able to include cases diagnosed by private gynaecologists as we do not have access to this information. Our results are therefore only applicable for women with a hospital-based diagnosis. In our study, we investigated healthcare utilization as a potential consequence of the diagnostic delay. However, we do not have information on the specific reason for contacting the GP, private gynaecologists and the hospitals, and can therefore only speculate that the differences found among cases and controls were related to endometriosis and the diagnostic delay. We included some paraclinical tests that might be provided by the GP on the account of endometriosis symptoms, but it is a limitation of this study that we lack information on the indication for providing the test and the result of the test.

We were able to adjust for several potential confounders, like region of residence where a previous study found that the probability of receiving an endometriosis diagnosis differed between the Danish regions (Illum *et al.*, 2022). However, unmeasured confounding might be a limitation in our study.

## Conclusion

In the present study, we found that women with endometriosis had an overall higher utilization of both primary and secondary healthcare in each of the 10 years before receiving a hospital-based endometriosis diagnosis, compared to women without diagnosed endometriosis.

To our knowledge, this is the first study to investigate the utilization of healthcare in a period corresponding to the estimated length of the diagnostic delay of endometriosis. The diagnostic delay of endometriosis is well known, but based on the results from this study, it does not seem to be due to a lack of contact with the healthcare system. Future studies should include the specific reasons for healthcare contacts to study if there are any specific contact patterns for women with endometriosis in the time before diagnosis. Hopefully, this could help healthcare professionals to be better at relating certain healthcare seeking behaviour to endometriosis earlier and thereby reduce the time from onset of symptoms to diagnosis.

## Data Availability

Due to restrictions related to Danish law and protecting patient privacy, the combined set of data as used in this article can only be made available through a trusted third party, Statistics Denmark. This state organization holds the data used for this study. University-based Danish scientific organizations can be authorized to work with data within Statistics Denmark and such organizations can provide access to individual scientists inside and outside of Denmark. Requests for data may be sent to Statistics Denmark.
